# Stellate nonheritable idiopathic foveomacular retinoschisis in juveniles: case report

**DOI:** 10.1186/s12886-023-03142-6

**Published:** 2023-09-26

**Authors:** Jianan Liu, Yanhui Wang, Lifei Wang

**Affiliations:** 1 Department of Ophthalmology, Hebei Medical University, Shijiazhuag, 050017 Hebei China; 2 Present Address: Hebei Eye Hospital, XingTai, Hebei China

**Keywords:** Juvenile, Vitrectomy, Stellate nonhereditary idiopathic foveomacular retinoschisis, Optical coherence tomography, Case report

## Abstract

**Background:**

Stellate nonhereditary idiopathic foveomacular retinoschisis (SNIFR) is a rare type of retinoschisis with a spoke-like splitting that occurs in the outer plexus layer. We present a case of stellate nonhereditary idiopathic foveomacular retinoschisis in a juvenile, in which two eyes show different development trends and macular retinoschisis could be associated with mechanical force in the Henle fibre layer. The removal of mechanical force can partially restore vision.

**Case presentation:**

A 14-year-old girl with bilateral SNIFR was diagnosed and followed up with spectral-domain optical coherence tomography (SD-OCT). During the two follow-up visits, vitreous adhesion was released in the left eye, and visual acuity improved. Neuroepithelial detachment was aggravated in the right eye, and visual acuity decreased. Therefore, vitrectomy was performed on the right eye. After surgery, the patient’s retina was reattached, and her vision was partially restored.

**Conclusions:**

We reported a juvenile with bilateral SNIFR. Each of her eyes showed different development trends, so we adopted different treatment methods for each eye. Vitrectomy was performed on the patient to address progressive vision loss, which improved the patient’s vision. It was further confirmed that the Henle layer of SNIFR patients was susceptible to posterior vitreous membrane adhesion.

**Supplementary Information:**

The online version contains supplementary material available at 10.1186/s12886-023-03142-6.

## Background

Stellate nonhereditary idiopathic foveomacular retinoschisis was proposed by Ober in 2014 [1]. There is no known predisposing factor, and most cases are unilateral, while a few are bilateral. At present, the diagnosis of SNIFR is determined after excluding other causes and there is no universal treatment.

We report a case of SNIFR in a juvenile. Visual acuity improved after the release of the vitreomacular adhesion in the left eye, and vitrectomy was performed in the right eye due to progressive visual acuity loss. The patient’s retina was **reattached**, and visual acuity improved after surgery.

## Case presentation

**Fig. 1:**
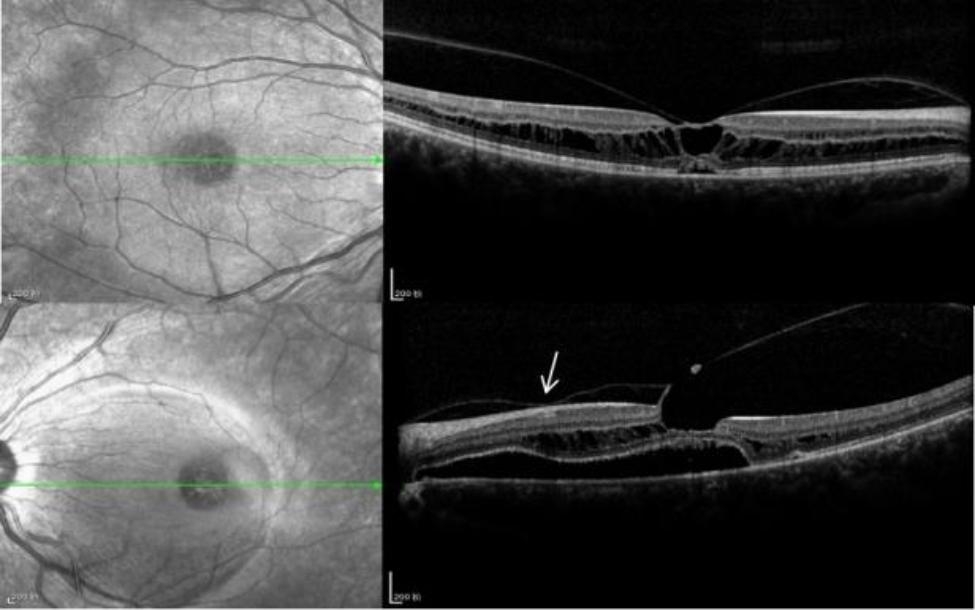
OCT images of both eyes when the patient’s firs’t visit to our hospital. **(a)** OD shows posterior Vitreous attachment in the macula (white arrow) with splits extending peripherally from the fovea. **(b)** OS shows that the outer retina of the left eye (Henle fibrous layer) is extensively split, and vitreous macular adhesion is mainly between the macula and optic disc, which is also the area of neuroepithelial detachment (white arrow)

**Fig. 2:**
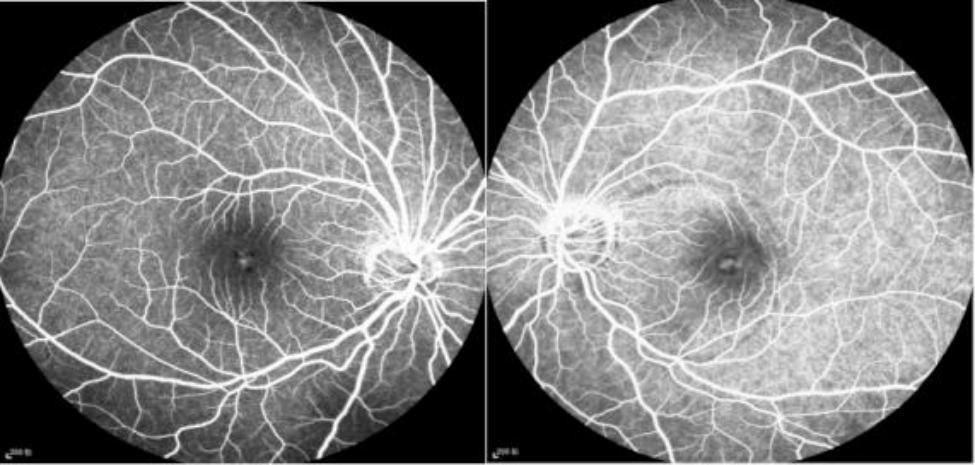
The patient’s FFA at the first visit showed macular split in both eyes

A 14-year-old girl came to our outpatient clinic with vision loss in both eyes for one year. She had previously been diagnosed with macular retinoschisis but had not been treated. Her visual acuity was checked when she first visited our hospital. Patient refraction was − 2.25/-1.50 × 155° in OD and − 2.50-/1.00 × 179° in OS. The best corrected visual acuity was **20/66** in OD and **20/100** in OS. On the SD-OCT scan, the OD presented a radial split in the fovea of the macular region with vitreomacular adhesion. The SD-OCT scan of the OS showed that the outer retina of the left eye (**Henle fiber layer**) was extensively split, and vitreous macular adhesion was mainly located between the macula and optic disc, which was also the area of neuroepithelial detachment (Fig. 1). Fundus fluorescein angiography (FFA )showed macular split in both eyes. Based on fundus microscopy and imaging, a diagnosis of macular retinoschisis was made (Fig. 2).

**Fig. 3:**
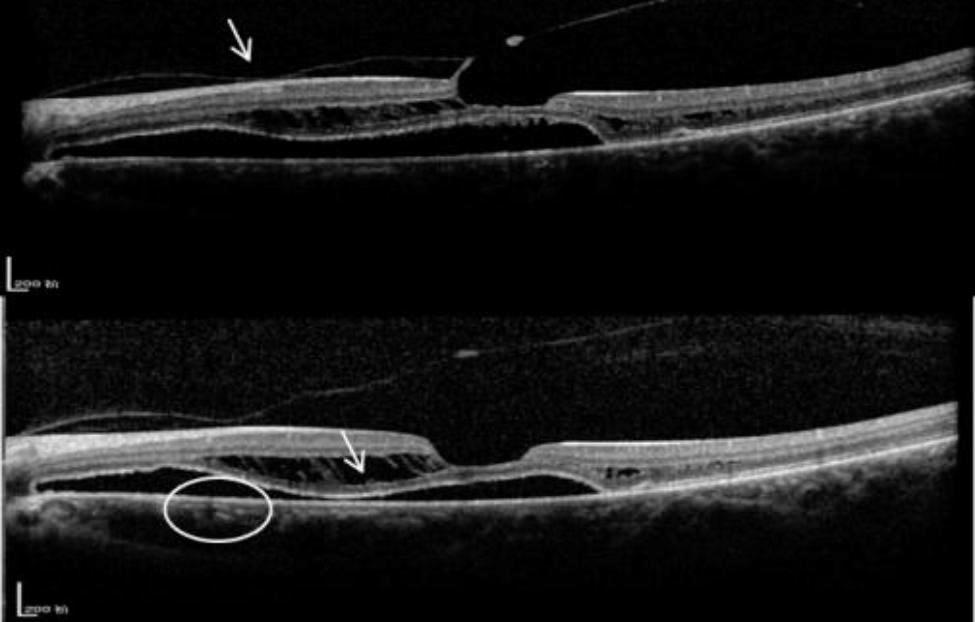
SD-OCT scan of OS. Vitreoretinal adhesion subsided and the detached neuroepitheliuml appeared to reset (posterior vitreous adhesion in white circle recedes)

**Fig. 4:**
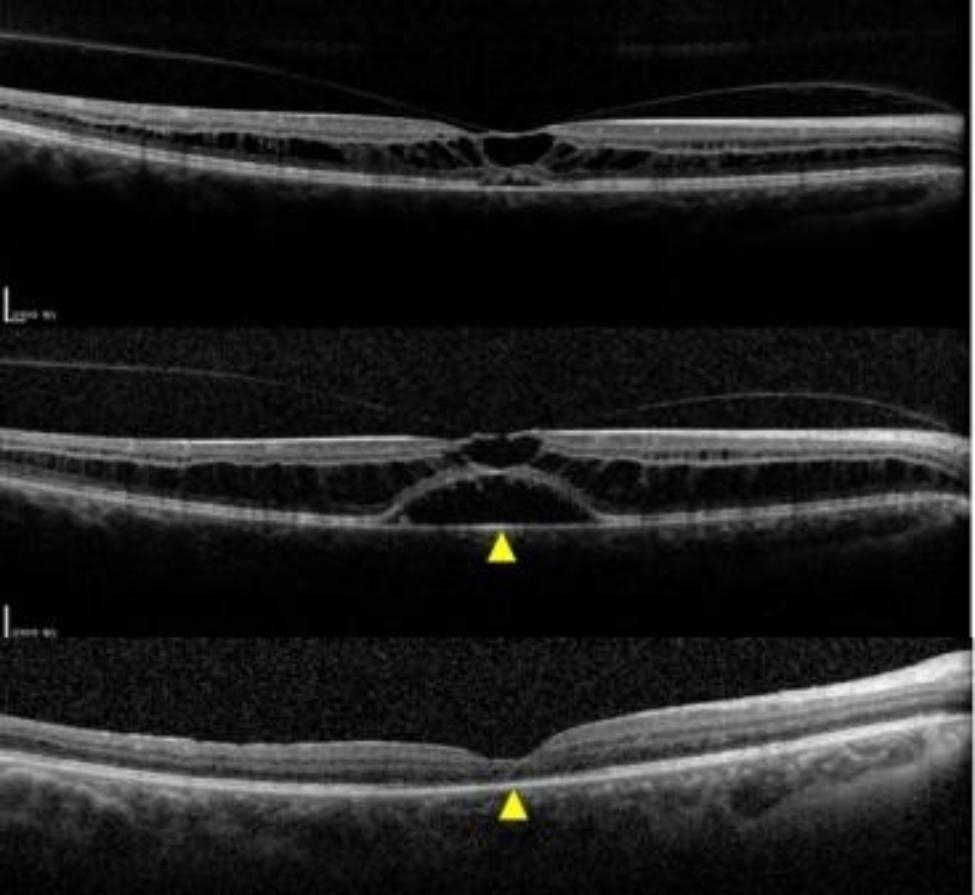
SD-OCT scan of OD. The degree of neuroepithelial detachment in the macular area increased after one month. **(a-b)** The retina of the patient’s OD was entirely attached after vitrectomy.**(b-c)**

To further identify the patient’s aetiology and determine whether surgery was needed, blood samples were taken for genetic testing with the patient’s consent. The results of the genetic test returned a negative match for the X-linked retinoschisis (CXLR) genotype, enhanced S-cone syndrome and Goldman-Favre syndrome, and the patient said she had no previous history of eye problems. The patient’s visual acuity in the right eye decreased from 20/66 to 20/80, and in the left eye increased from 20/80 to 20/50 during the month of waiting for test results. An SD-OCT scan of the OS showed that vitreoretinal adhesion subsided, and the detached neuroepithelium appeared to reset, which may explain the improvement in Best Corrected Visual Acuity(BCVA) (Fig. 3). However, the degree of neuroepithelial detachment in the macular area of the right eye was increased (Fig. 4).

According to the results of the patient’s genetic test report and clinical manifestations, we believe that it is unlikely that this patient had CXLR. The presence of an optic disc pit was ruled out by a high-density raster pattern of enhanced depth imaging SD-OCT, and the patient denied previous use of **niacin** or **taxane-derived therapy**. Clinical examination ruled out pathological myopia, glaucoma, myopic traction macular disease, enhanced S-cone syndrome and other retinopathies, and the patient was diagnosed with SNIFR. Since the vitreous adhesion of the patient’s left eye was removed and the visual acuity was restored to some extent, we only performed vitrectomy to remove the vitreous adhesion of the right eye, where visual acuity loss occurred, in the hope of improving the patient’s vision. The patient’s consent was obtained at the same time.After surgery, the patient’s retina was **reattached** (Fig. 4).The postoperative visual acuity of the patient was **20/66** in OD,**20/33** in OS.

## Discussion and conclusions

Stellate nonhereditary idiopathic foveomacular retinoschisis was first described by Ober in 2014 as a new category of macular retinoschisis [1]. 94% of the SNIFR patients are female. All affected patients presented with foveal cleavage, and nearly half developed peripheral cleavage. These splits mainly occurred in the outer plexus layer in the macula. 86% of patients had an attached posterior hyaloid, and visual acuity was relatively maintained (≥ 20/40). However, subretinal fluid development under the fovea seriously affects visual acuity.

While previous cases have only been reported in adults, this is the first known report of SNIFR in a minor. Moreover, the two eyes of the patient presented different visual changes due to different release trends of adhesions. From the SD-OCT of the left eye, we observed the release of vitreous adhesions between the macula and optic disc. The detached neuroepithelium showed some reduction in eyesight. This suggest that vitreous adhesion release is associated with remission of SNIFR, which is consistent with previous reports. Edward Bloch found spontaneous improvement of fovea splitting in two subjects with posterior vitreous separation [2]. Nogueira [3] also reported a case of SNIFR regression after the release of vitreous adhesion. In this case, the patient was younger, which suggests that the patient may have congenital abnormalities in the vitreo-retinal interface. In the case of vitreo-retinal interface adhesion, the retina may be more susceptible to the influence of adhesion factors, leading to splits.

The pathogenesis of this disease remains unclear. OCT images of patients with SNIFR were “spoke-wheel”, and splitting occurred in the outer plexiform layer/Henle fibrous layer, with nearly normal INL thickness, which is a manifestation of retinal tension. It was previously stated that the preferred location of schisis in the retina is the Henle fibrous layer because the horizontal processes of parafoveal Z-shaped Müller cells are located in this layer. Mechanical forces may physically separate and displace Müller cell processes in the HFL [4]. In this model, the “spokes” represent the Henle fibres that show high reflection in OCT images. Therefore, it is speculated that cleft formation in SNIFR patients is affected by mechanical force, causing disruption of fluid balance in the retinal interstitium. The formation and maintenance of the retinal cystic space are closely related to mechanical force, and the removal of mechanical force is conducive to the reabsorption of interstitial fluid by the Retinal pigment epithelium(RPE) pump and Muller cells. Finally, the split retinal structure can recover slowly. Vitrectomy was performed to remove the adhesion between the vitrectomy and retina, and the split and detached retinal structures were restored after surgery as expected. Other authors have performed vitrectomy in SNIFR patients with severe vision loss, and the patient’s vision recovered from 20/100 to 20/33 after surgery [5]. Combined with the leading hypothesis that the pathogenesis of SNIFR is related to abnormalities in the vitreo-macular interface or traction factors, vitrectomy can be used to treat patients with progressive vision loss and no tendency of vitreous adhesion release.

In conclusion, cases of juvenile patients may support the view that patients with SNIFR have congenital vitreo-macular interface abnormalities or that the Henle fibre layer is more susceptible to traction factors to produce splits. In patients with progressive vision loss, vitrectomy to remove adhesion factors may be beneficial to improve vision.

### Electronic supplementary material

Below is the link to the electronic supplementary material.

Supplementary Material 1

## Data Availability

All data generated or analyzed during this study are included in this published article and its supplementary information files.

## Declarations

All methods were performed in accordance with the ethical standards as laid down in the Declaration of Helsinki and its later amendments or comparable ethical standards.

Written informed consent was obtained from all participants (Participant and her legal guardian.). Include a statement on ethics approval and consent.

## List of abbreviations

SNIFR: Stellate nonhereditary idiopathic foveomacular retinoschisis
SD-OCT: Spectral-domain optical coherence tomography
CXLR: X-linked retinoschisis
FFA: Fundus fluorescein angiography
