# Supplementary material for: Stellate nonheritable idiopathic foveomacular retinoschisis in juveniles: case report
Source: BMC Ophthalmol. 2023 Sep 26;23:389. doi: 10.1186/s12886-023-03142-6 (PMC10521493; doi:10.1186/s12886-023-03142-6)
Supplement: Supplementary file 1 — Supplementary Material 1 [file 12886_2023_3142_MOESM1_ESM.pdf]

CARE case report guidelines

CARE Checklist of information to include when writing a case report

(cc) BY-NC-ND

✓

Topic	Item	Checklist item description	Reported on Line
Title	1	The diagnosis or intervention of primary focus followed by the words "case report"	Title，page1，1、2
Key Words	2	2 to 5 key words that identify diagnoses or interventions in this case report, including "case report"	KeyWords，page2，31、32
Abstract (no references)	3a	Introduction: What is unique about this case and what does it add to the scientific literature?	Conclusions，page2
	3b	Main symptoms and/or important clinical findings	Case presentation，page3
	3c	The main diagnoses, therapeutic interventions, and outcomes	Case presentation，page3
	3d	Conclusion—What is the main "take-away" lesson(s) from this case?	Conclusions，page2
Introduction	4	One or two paragraphs summarizing why this case is unique (**may include references**)	Discussion and conclusions，page5
Patient Information	5a	De-identified patient specific information.	Case presentation，page3
	5b	Primary concerns and symptoms of the patient.	Case presentation，page1
	page	Medical, family, and psycho-social history including relevant genetic information	Case presentation，page4，70
	5d	Relevant past interventions with outcomes	Case presentation，page3，46
Clinical Findings	6	Describe significant physical examination (PE) and important clinical findings.	Case presentation，page3，51-58
Timeline	7	Historical and current information from this episode of care organized as a timeline	Case presentation，page3
Diagnostic Assessment	8a	Diagnostic testing (such as PE, laboratory testing, imaging, surveys).	Case presentation，page3
	8b	Diagnostic challenges (such as access to testing, financial, or cultural)	Case presenation，page4
	8c	Diagnosis (including other diagnoses considered)	Case presenation，page4，75
	8d	Prognosis (such as staging in oncology) where applicable	Case presentation，page4
Therapeutic Intervention	9a	Types of therapeutic intervention (such as pharmacologic, surgical, preventive, self-care)	Case presenation，page4，76、77
	9b	Administration of therapeutic intervention (such as dosage, strength, duration)	Case presenation，page4
	9c	Changes in therapeutic intervention (with rationale)	Discussion and conclusions，page6，127
Follow-up and Outcomes	10a	Clinician and patient-assessed outcomes (if available)	
	10b	Important follow-up diagnostic and other test results	Case presenatio page4.
	10c	Intervention adherence and tolerability (How was this assessed?)	Case presenatio page4
	10d	Adverse and unanticipated events	
Discussion	11a	A scientific discussion of the strengths AND limitations associated with this case report	Discussion and conclusions,page5
	11b	Discussion of the relevant medical literature **with references**.	Discussion and conclusions,page5,97
		The scientific rationale for any conclusions (including assessment of possible causes)	Discussion and conclusions,page5
	11d	The primary "take-away" lessons of this case report (without references) in a one paragraph conclusion	Discussion and conclusions,page6
Patient Perspective	12	The patient should share their perspective in one to two paragraphs on the treatment(s) they received	Case presentation,page4,79
Informed Consent	13	Did the patient give informed consent? Please provide if requested	Yes ☑ No ☐
